# Structural Abnormalities in the Hair of a Patient with a Novel Ribosomopathy

**DOI:** 10.1371/journal.pone.0149619

**Published:** 2016-03-16

**Authors:** Richard J. Alsop, Asfia Soomro, Yuchen Zhang, Marc Pieterse, Ayodele Fatona, Kimberly Dej, Maikel C. Rheinstädter

**Affiliations:** 1 Department of Physics and Astronomy, McMaster University, Hamilton, Ontario, Canada; 2 Department of Chemistry and Chemical Biology, McMaster University, Hamilton, Ontario, Canada; 3 Department of Biology, McMaster University, Hamilton, Ontario, Canada; Massey University, NEW ZEALAND

## Abstract

We report the biophysical characterization of hair from a patient with a *de novo* ribosomopathy. The patient was diagnosed with a mutation on gene RPS23, which codes for a protein which comprises part of the 40S subunit of the ribosome. The patient presents with a number of phenotypes, including hypotonia, autism, extra teeth, elastic skin, and thin/brittle hair. We combined optical microscopy, tensile tests, and X-ray diffraction experiments on hair samples obtained from the scalp of the patient to a multi-scale characterization of the hair from macroscopic to molecular length scales and observe distinct differences in the biophysical properties in the patient’s hair when compared to hair from other family members. While no differences were observed in the coiled-coil structure of the keratin proteins or the structure of the intermediate filaments, the patient’s hair was 22% thinner, while the Young’s modulus remained roughly constant. The X-ray diffraction results give evidence that the amount of lipids in the cell membrane complex is reduced by 20%, which well accounts for the other observations. The pathologies characterized by these techniques may be used to inform the diagnosis of similar *de novo* mutations in the future.

## Introduction

### Clinical Pathologies from *de novo* Diseases

A clinical challenge often exists in addressing diseases arising from *de novo* mutations, as there is no defined diagnostic or treatment protocol. Some disease may present with symptoms similar to a different disease, leading to mis-diagnosis. Exome sequencing is rapidly becoming a useful tool to determine the genetic causes of *de novo* diseases [1]. However, exome sequences may identify a number of genetic variants, which can add confusion when trying to determine the source of disease pathologies. The fast and detailed characterization of clinical pathologies can be a useful exercise when treating patients with *de novo* diseases and understanding their exomes [1], as only 10-50% of exome sequences result in a diagnosis [2, 3].

Here, we report the biophysical characterization of hair obtained from a Dutch, 12-year old, male patient with a *de novo* ribosomopathy. After 3 years of work, the patient was diagnosed with a mutation on gene RPS23, which codes for a protein which comprises part of the 40S subunit of the ribosome. The patient presents with a number of phenotypes, including hypotonia, autism, extra teeth, elastic skin, and thin/brittle hair. While the RPS23 gene mutation is associated with the patient’s ribosomopathy, there is no clear connection between the mutation and the pathologies.

Optical microscopy, tensile tests, and X-ray diffraction experiments were performed on hair samples obtained from the scalp of the patient and also hair from his family. These tests allowed for a multi-scale characterization of the hair from macroscopic to molecular length scales. We observe distinct differences in the biophysical properties in the patient’s hair when compared to hair from other family members.

### General Properties of The Hair Fibre

**Fig 1:**
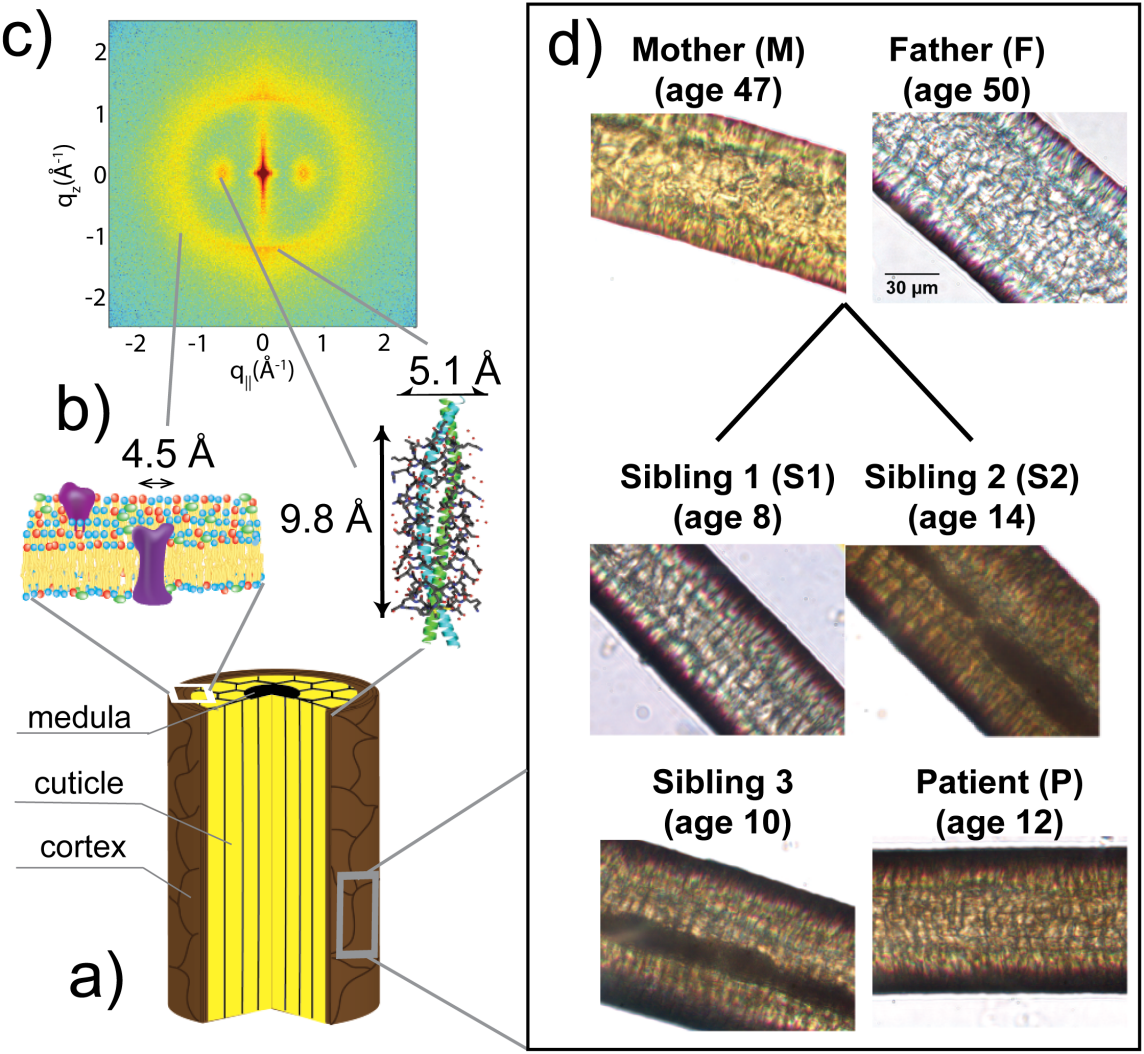
Illustration of the structure of hair. a) Sketch of a human hair showing the three main regions, the *medulla*, the *cuticle*, and the *cortex*. b) Molecular cartoons of hair structures: keratin coiled-coil dimers and lipids in the cell membrane complex. c) A sample 2-dimensional X-ray diffraction image, indicating the assignment of structures to signals, as outlined in the Results. d) Optical microscopy images of the hair surface of all persons involved in this study.

Human hair is a layered structure with a total diameter between 40-110 *μ*m (Fig 1a and 1b). The inner most layer is the *medula*, followed by the *cortex*, then the *cuticle*, all bound together by cell-membrane complexes. The *medula* is a thin structure composed largely of disordered protein. The majority of the hair fibre is the *cortex*, which contains spindle shaped cells that lie parallel along the fibre axis. Most cortical cells are composed of keratin protein that is an *α*-helical protein, which forms coiled-coil dimers in strands along the fibre axis. 7 such dimers form bundles 75-90 Å in diameter, known as protofilaments. These protofilaments further aggregate into intermediate filaments. The *cuticle* is the outermost layer, which is composed of overlapping dead cells that form a protective barrier against the outside environment [4].

The details of hair structure and organization have been studied in the past with X-ray diffraction. In particular, the *α*-helical keratin structure within the *cortex* has been extensively studied since the 1930’s [5–9]. A typical diffraction pattern is shown in Fig 1c).

X-ray diffraction observes signals from keratin in protofilament bundles, the coiled-coil keratin dimers, and the lipids in the cell membrane complex. We have previously used X-ray diffraction to study the hair of a number of individuals with differing characteristics. Genetic similarities were observed, *i.e.*, similarities between the patterns observed in the hair of father and daughter strands, as well as similarities in identical twins [8]. We have also used X-ray diffraction to probe the effect of hair treatments on the hair structure. While no change was observed from the single use of shampoo, structural changes were observed due to permanent waving treatment [9].

Changes to the molecular structure of the fibre will likely translate into changes in mechanical properties. Tensile tests of hair were performed by measuring the force required to pull the hair along its long axis. From the resultant stress-strain curve, the Young’s modulus of the fibre was obtained.

Certain genetic disorders may be diagnosed based on structure and properties of the hair fibre. For example, in maple syrup urine disease (MSUD), the absence of the lipid 18-methyleicosanoic acid from the hair, caused by a mutation leading to the removal of an enzyme, comprimises the stability of the hair [10]. The development of a breast cancer screening protocol using X-ray diffraction of human hair has been extensively studied by James and Corino [11, 12]. This screening protocol is already in the clinical trial stage, with an overall accuracy of greater than 77% and a negative predictive value of 99% [12]. Here, we examine the physical appearance, structural integrity, and microscopic structure of hair to characterize a phenotype of a novel ribosomopathy.

## Results

### General Properties of the Hair Samples

This research was approved by the Hamilton Integrated Research Ethics Board (HIREB) under approval number 14-474-T. Written consent was obtained from all participating adults, and informed consent was also obtained, in writing, from the next of kin on behalf of the children enrolled in the study. The subjects of the study included six members of a Dutch family: the male subject or patient (aged 12 years, denoted P), his mother (47 years, denoted M); his father (50 years, denoted F); a sister, denoted as Sibling 1 (8 years, S1); a brother, denoted as S2 (14 years, S2); and a second sister, herein denoted as S3 (10 years, S3). A set of ∼50 hairs were received, untreated, from each person. Upon initial visual inspection, all hair samples appeared normal, with no obvious deformities. Some hairs from subject F were grey, likely due to his age [4, 13]. The family maintains standard Western hygiene practices, and uses shampoo and conditioner on a regular basis such that the hairs cannot be considered “virgin hairs” [4].

Surface images obtained of hair samples from all subjects were obtained using an Inverted Microscope. Representative images are shown in Fig 1d). The surface of all hair samples appear normal, with characteristic overlapping dead-cells forming a shingle-like appearance [4]. No hairs imaged appeared to have surface abnormalities.

**Fig 2:**
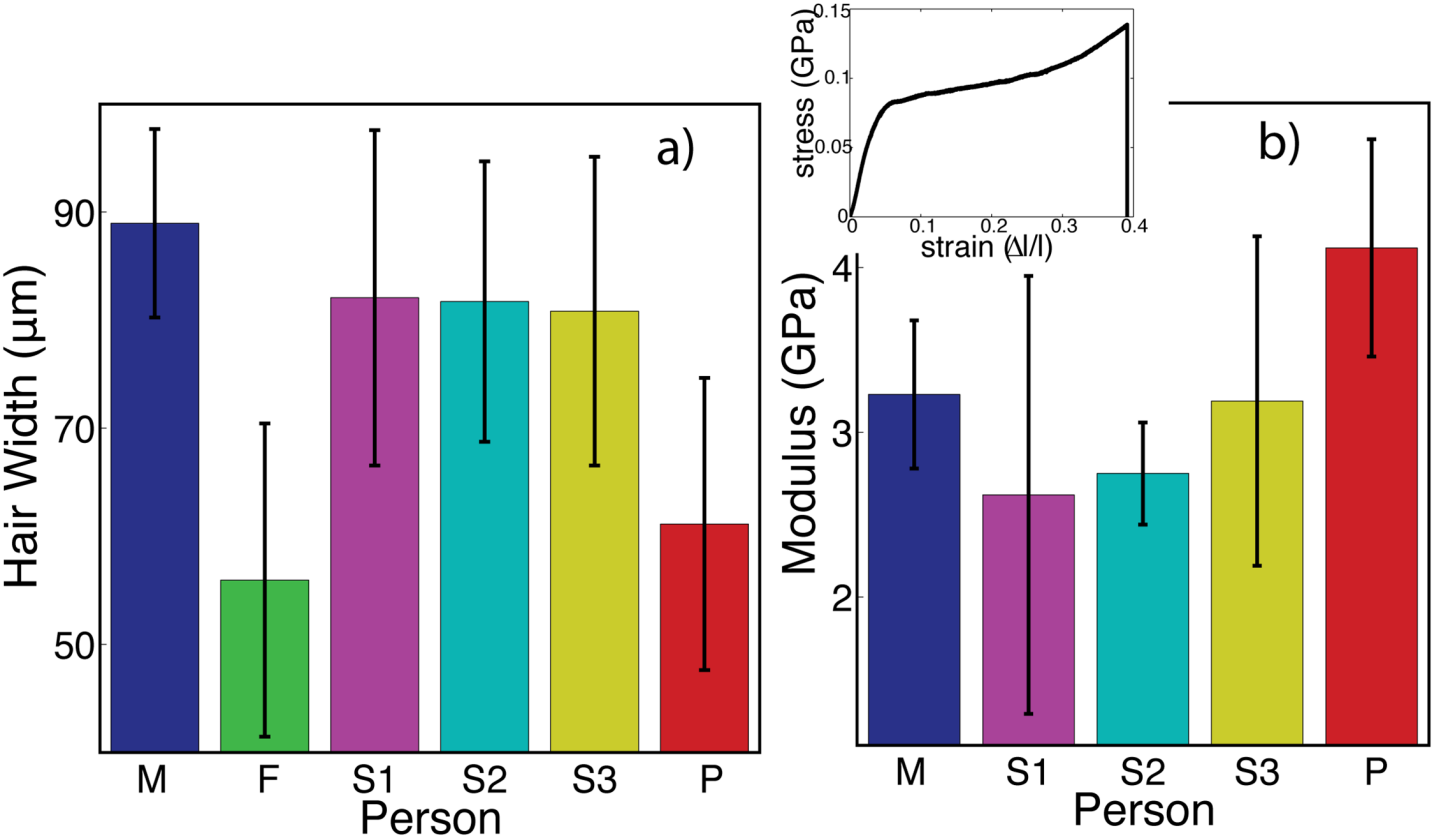
Results of microscopy and hair experiments. a) The average diameter of the hair samples. Diameters were measured in the centre of the hair fibre. Ten hairs were measured per person and averaged. b) The Young’s modulus of hair from different individuals, measured from tensile tests. The modulus from three hairs were measured and averaged (with the exception of S3). Note that hairs from F were too short to measure with the setup used. The inset in b) shows a typical stress-strain curve. Error bars in both plots represent the standard deviation within the measurements.

The diameter of select hairs from each individual was extracted from microscope images, as described in Materials and Methods. Hair widths were measured in the centre of the hair fibre. Ten hairs were measured for each subject, and the averages and standard deviations are displayed in Fig 2a). The average hair diameters were between 50 *μ*m and 90 *μ*m, with standard deviations on the order of 10 *μ*m, in agreement with previous reports [13–16]. While subjects M, S1, S2, and S3 have average hair diameters of 80-90 *μ*m, subjects F and P have diameters between 50-60 *μ*m, indicative of a decrease of ∼25%.

### Results of Tensile Tests

Tensile tests were performed to examine the stiffness of the hair samples, as described in Materials and Methods. Three hairs from each person were tested, with a single measurement per hair. Stress-strain curves were obtained, which describe the force needed to extend a hair of length ℓ to an extension of Δℓ, recorded from Δℓ = 0 up to the point of hair fracture. A sample curve is shown in Fig 2b) (inset). The first linear portion of the curve is related to elastic stretching of keratin in the hair fibre, and the slope of this region is proportional to the Young’s modulus of the fibre [17, 18].

The corresponding elastic moduli are plotted in Fig 2b), along with the mean and standard deviation measured for each person. Note that F is not represented in the pulling experiments as his hairs were too short to be accurately measured with the setup available. Also, only two measurements are shown for S2 as the third yielded a result which was an order of magnitude higher than the rest, suggesting a systematic error occured during this measurement. The elastic moduli measured with this analysis are between 2 GPa and 6 GPa, which agree well with previous reports for human hair [16]. While the hair from the patient appears slightly stiffer than the others (4.1±0.7 compared to 3±0.5), the difference is not statistically significant.

### Diffraction Analysis

**Fig 3:**
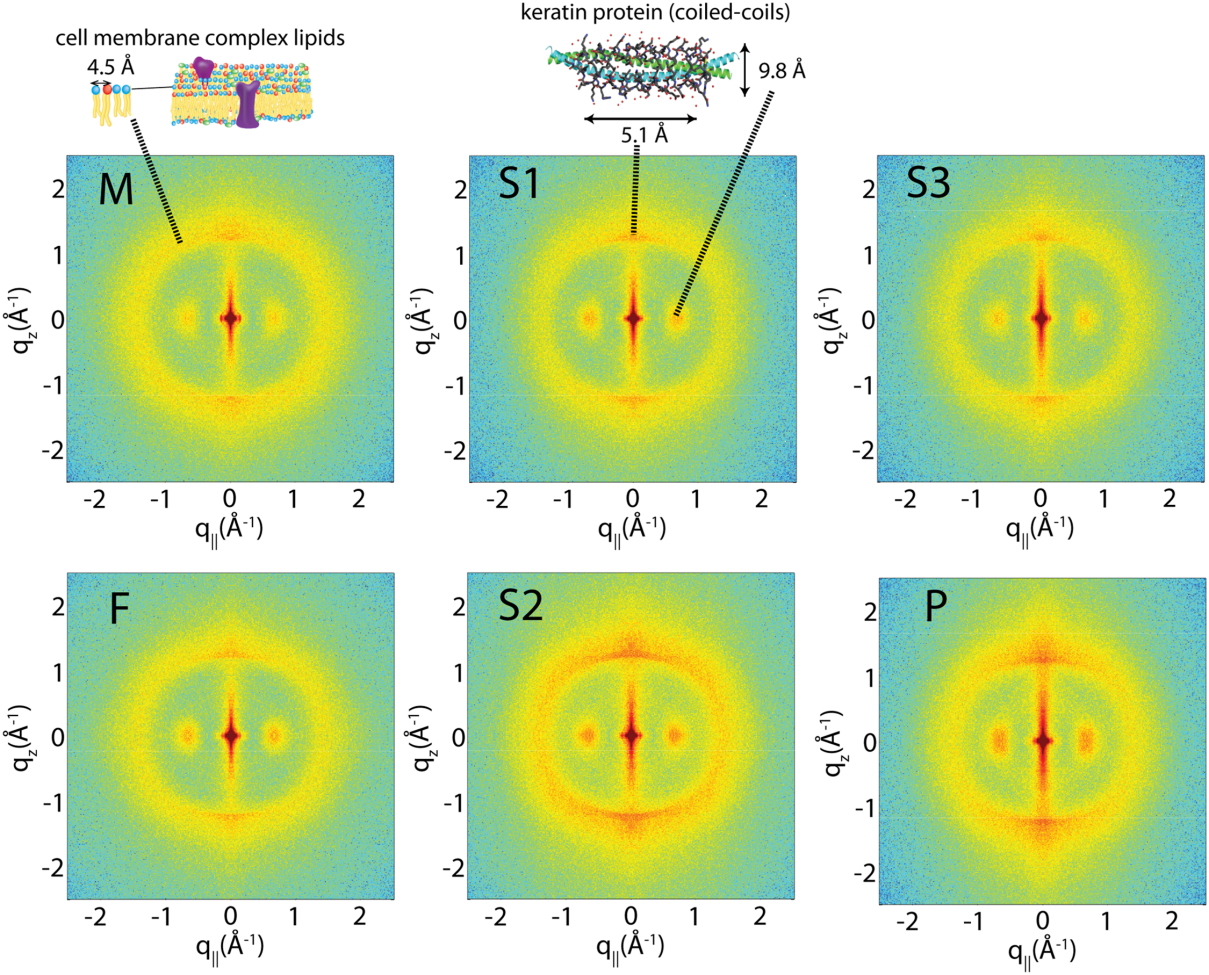
Two-dimensional X-ray diffraction images from all hair samples. The hair strands were oriented with the long axis of the hair parallel with the vertical *z*-axis. The displayed (*q*_*z*_, *q*_||_) range covered length scales from 3 Å up to 250 Å, to study the coiled-coil *α*-keratin phase, as well as the membrane layer in the cortex [8, 9]. The features observed are common among the individuals in this study, and agree with previous reports.

Two-dimensional X-ray intensity maps were collected for hair samples from all individuals to search for differences in the molecular structure of the hair fibre, and are displayed in Fig 3. The hair strands were oriented such that their long axis was parallel with the vertical *z*-axis, and the displayed (*q*_*z*_, *q*_||_)-range was designed to cover the features of interest, as described in Materials and Methods. These maps exhibit the characteristic features observed previously [8, 9].

A number of peaks are observed and the assignment of scattering signals to biological structures is depicted in Fig 3. The keratin proteins in the *cortex* are known to organize in bundles, whose structure is dominated by *α*-helical coiled-coils [8, 21]. Coiled coils consist of *α*-helices wound together to form a ropelike structure stabilized by hydrophobic interactions. The coiled-coil motif is found in about 10% of the proteins in the human genome [22].

The main features of this motif is a ∼9.8 Å (*q*_||_∼0.66 Å^−1^) equatorial reflection corresponding to the spacing between adjacent coiled-coils and a ∼5.0 Å meridional reflection (*q*_*z*_∼1.25 Å^−1^) corresponding to the superhelical structure of *α*-helices twisting around each other within coiled-coils [23–25]. These signals were observed in the X-ray data. In addition, a broad ring of scattering was observed at *Q*∼ 1.6 Å^−1^ by scattering contributions from the membrane complexes and an amorphous halo, as will be discussed below. In the 2-dimensional data, scattering signals from all individuals show good agreement in signal position and shape, suggesting that the overall molecular structure of the hair is not different in the patient *vs.* the rest of the family.

**Fig 4:**
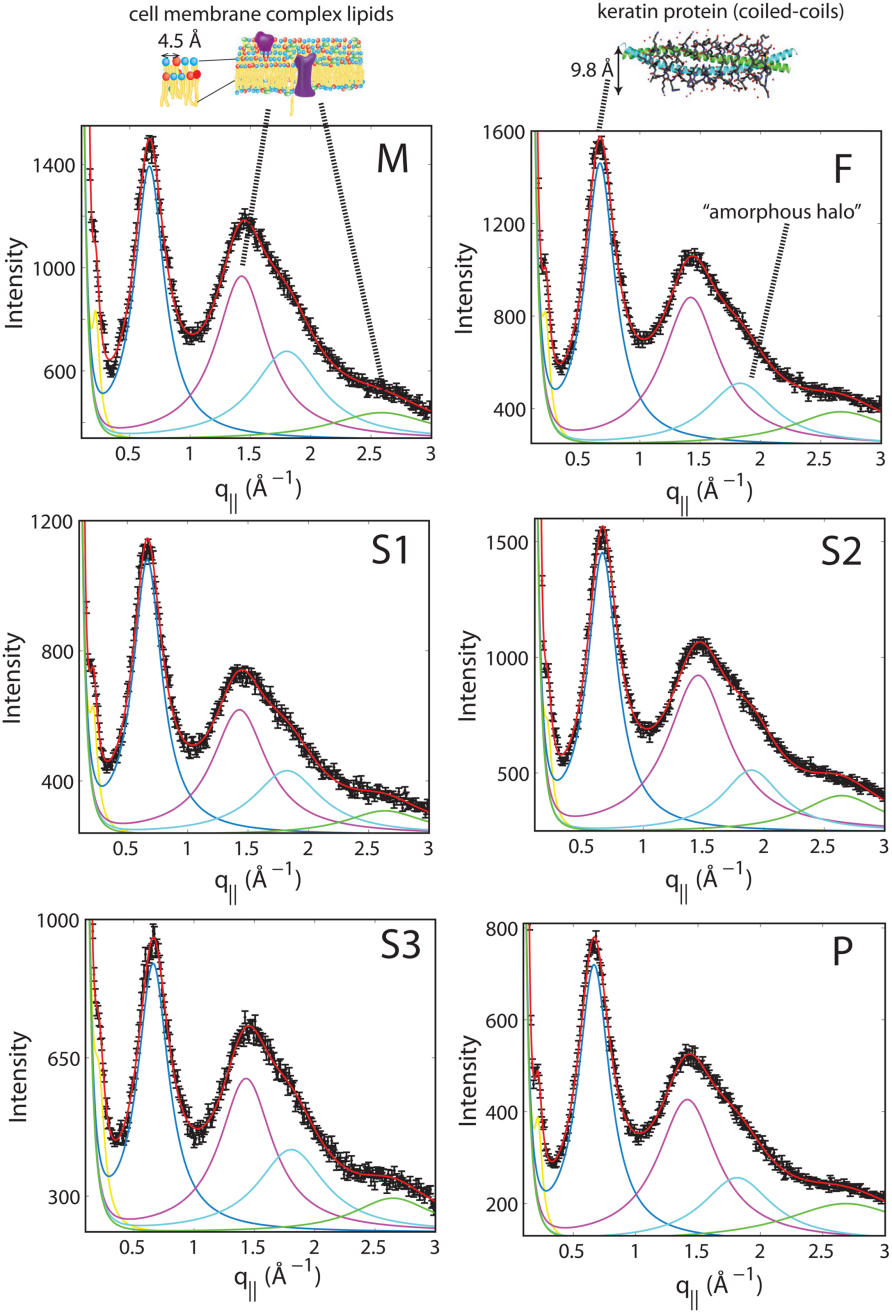
High resolution scans were measured from 0.2 Å^−1^ < *q*_||_ < 3.0 Å^−1^. Five peaks describe these scans, with an exponential background: i) a peak at 45 Å (yellow), assigned to keratin bundles and observed in SAXS profiles (Fig 6); ii) A peak at 9.8 Å, assigned to lateral packing of keratin coiled-coils; iii) A lipid peak at 4.5 Å; iv) A peak at 3.5 Å, assigned to amorphous protein; and v) A second lipid peak at 2.3 Å. No significant differences are observed in peak position or width, as listed in Table A in S1 File [8, 9, 19].

To better understand the observed scattering, and to check for the possibility of subtle changes in the molecular structure of the hair, high resolution line scans were recorded along *q*_||_, and displayed in Fig 4. These scans captured length scales of 2.1 Å up to 21 Å, and were performed with counting times 8-times longer than single lines in the 2-dimensional plots. Longer counting times significantly increased counting statistics. Lorentzian fits were used to describe the data. The profile at 9.8 Å represents scattering from keratin fibres. In addition, three other peaks were observed at 4.5 Å, 3.5 Å, and 2.3 Å.

**Fig 5:**
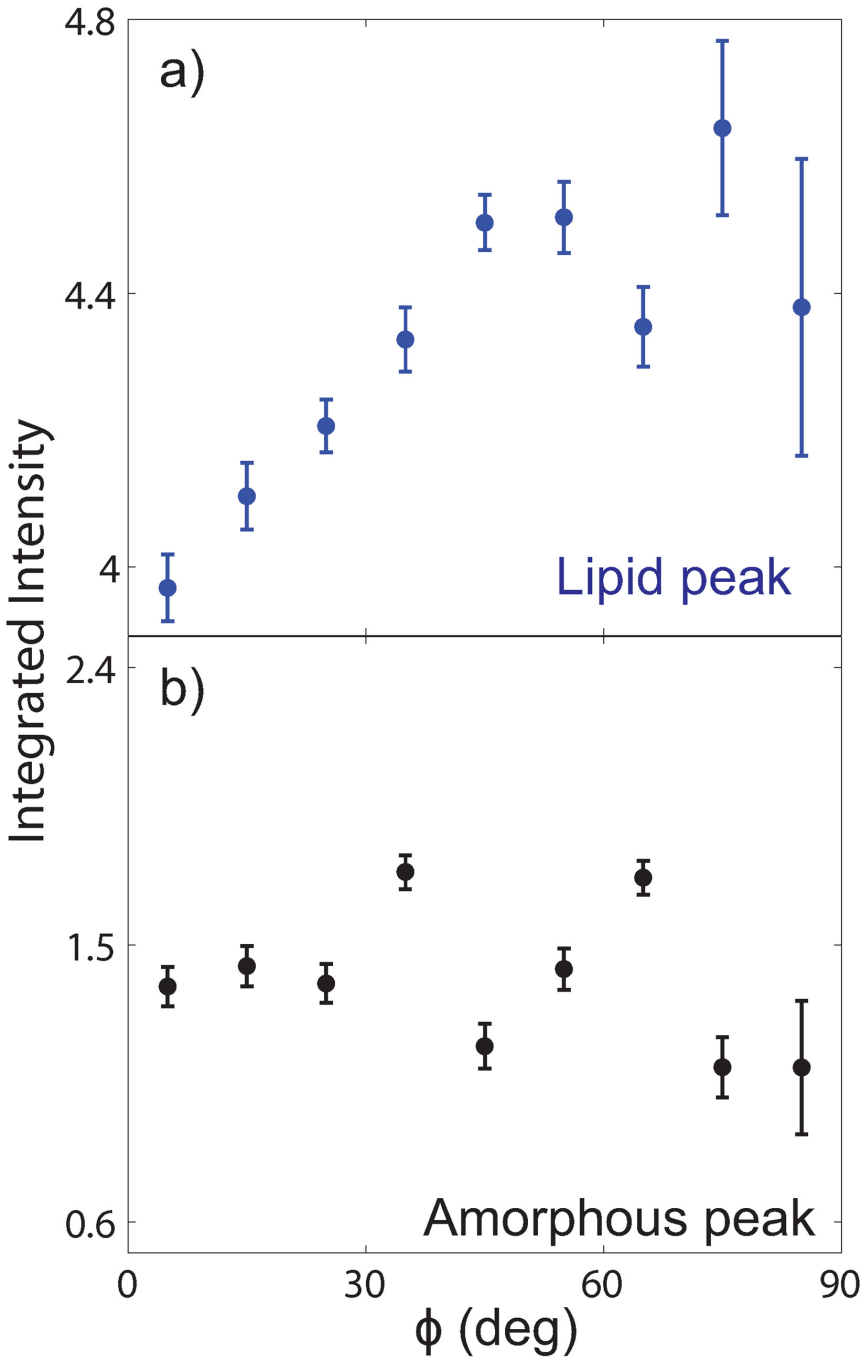
Angular distribution of scattering at the lipid and amorphous position. Peaks observed at a) *Q* = 1.40 Å^−1^ and b) *Q* = 1.80 Å^−1^ were integrated as a function of azimuth angle *ϕ* from the *q*_||_ axis, for subject M. While the peak at *Q* = 1.80 Å^−1^ is isotropic in *ϕ*, the peak at *Q* = 1.40 Å^−1^ is anisotropically distributed with a maximum in intensity at *ϕ* = 90°.

Peaks at 4.7 Å and 2.3 Å agree well with lipid correlation peaks observed in single and multi-component fluid lipid membranes [19, 26–29]. In particular, the appearance of a second order tail correlation peak at 2.3 Å is strong evidence this scattering can be assigned to ordered lipids within the cell membrane complex. A peak at 3.5 Å is a characteristic “amorphous halo” often observed in disordered polymer/biopolymer systems, and related to a typical carbon-carbon distance [30, 31]. However, as the cell membrane complex is oriented along the radial axis of the fibre, the lipid peak should be oriented along *q*_*z*_. The peaks at 4.5 Å and 3.5 Å were integrated as a function of azimuth angle *ϕ* away from the *q*_||_ axis from the 2-dimensional data and the results (for subject M) are displayed in Fig 5. The peak at 4.5 Å shows a maximum in intensity at *ϕ* = 90° in Fig 5a), while the peak at 3.5 Å in Fig 5b) is isotropic in *ϕ*. Therefore, we confidently assign scattering at 4.5 Å to lipids within the cell membrane complex.

Note that, within the resolution of the experiment, no changes were observed in the peak positions or widths among any of the hair samples tested, as shown in Table A in S1 File. The molecular structure of the hair does not appear to be different between individuals within the resolution of the current experiment.

**Fig 6:**
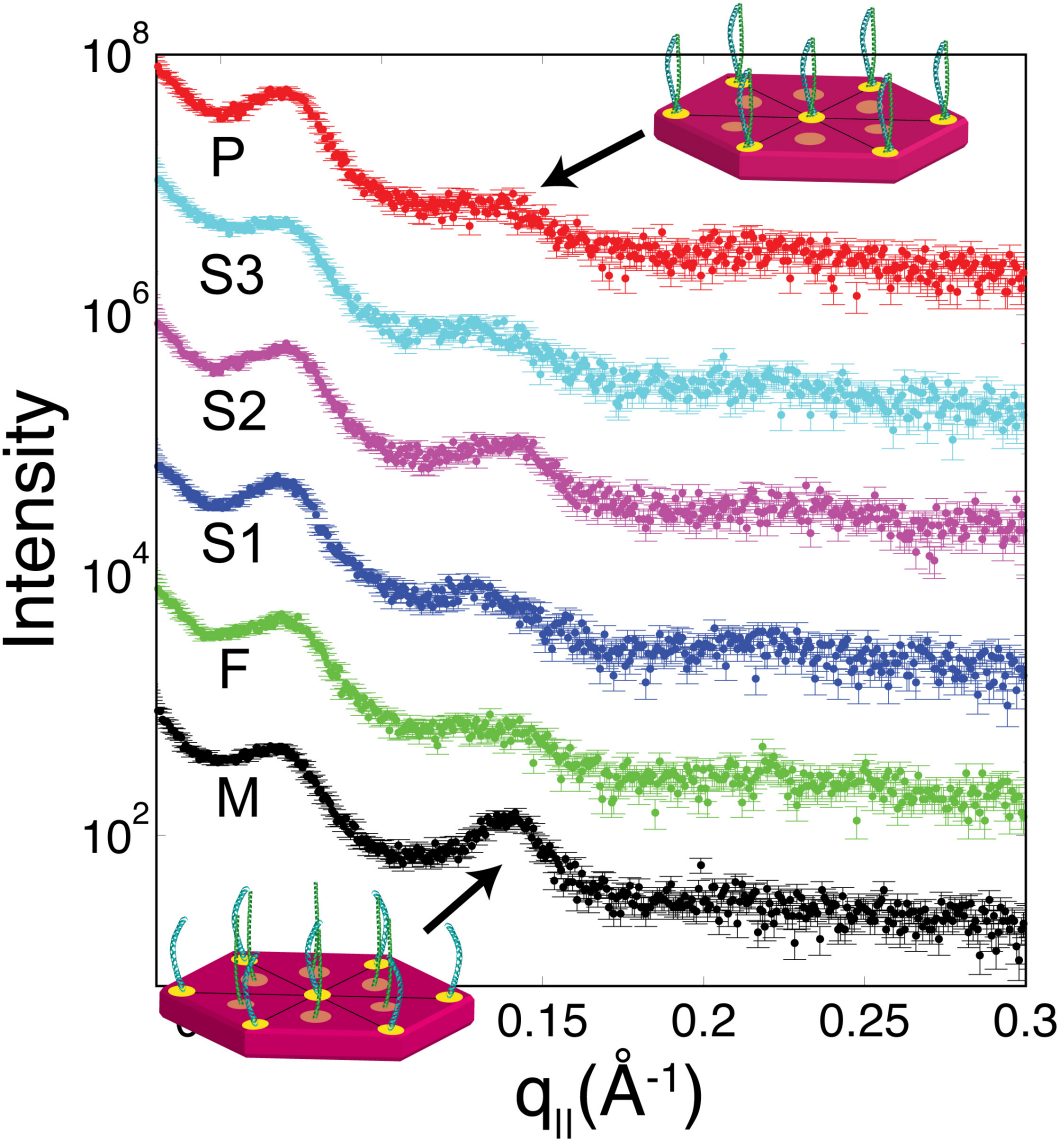
Small angle X-ray scattering profiles, covering length scales of 21 Å up to 250 Å. Peaks at 90 Å, 45 Å, and 27 Å are assigned to the structure of keratin bundles. Cartoons depict the structure of the keratin bundles: A hexagonal packing of keratin dimers for hairs from F, S1, S2, S3, and P, and a hexagonal packing of keratin monomers for M. The difference in packing between M and the others likely comes from the use of hair dyes or perming [4, 9, 20].

Small-angle X-ray scattering (SAXS) was performed along *q*_||_ from 0.03 Å^−1^ < *q*_||_ < 0.3 Å^−1^, covering length scales of 21 Å up to 250 Å. Resultant curves are shown in Fig 6. SAXS profiles of human hair show peaks at 90 Å, 45 Å, and 27 Å, which describe the form-factor of keratin protofilaments. No changes in position or width in any of these peaks are observed among the subjects tested. However, an observable increase in intensity is observed at 45 Å for M. In a recent X-ray diffraction study of human hair, an increase in this peak was shown to be the result of permanent waving treatment, caused by the use of reducing/oxidating agents in the treatment [9].

**Fig 7:**
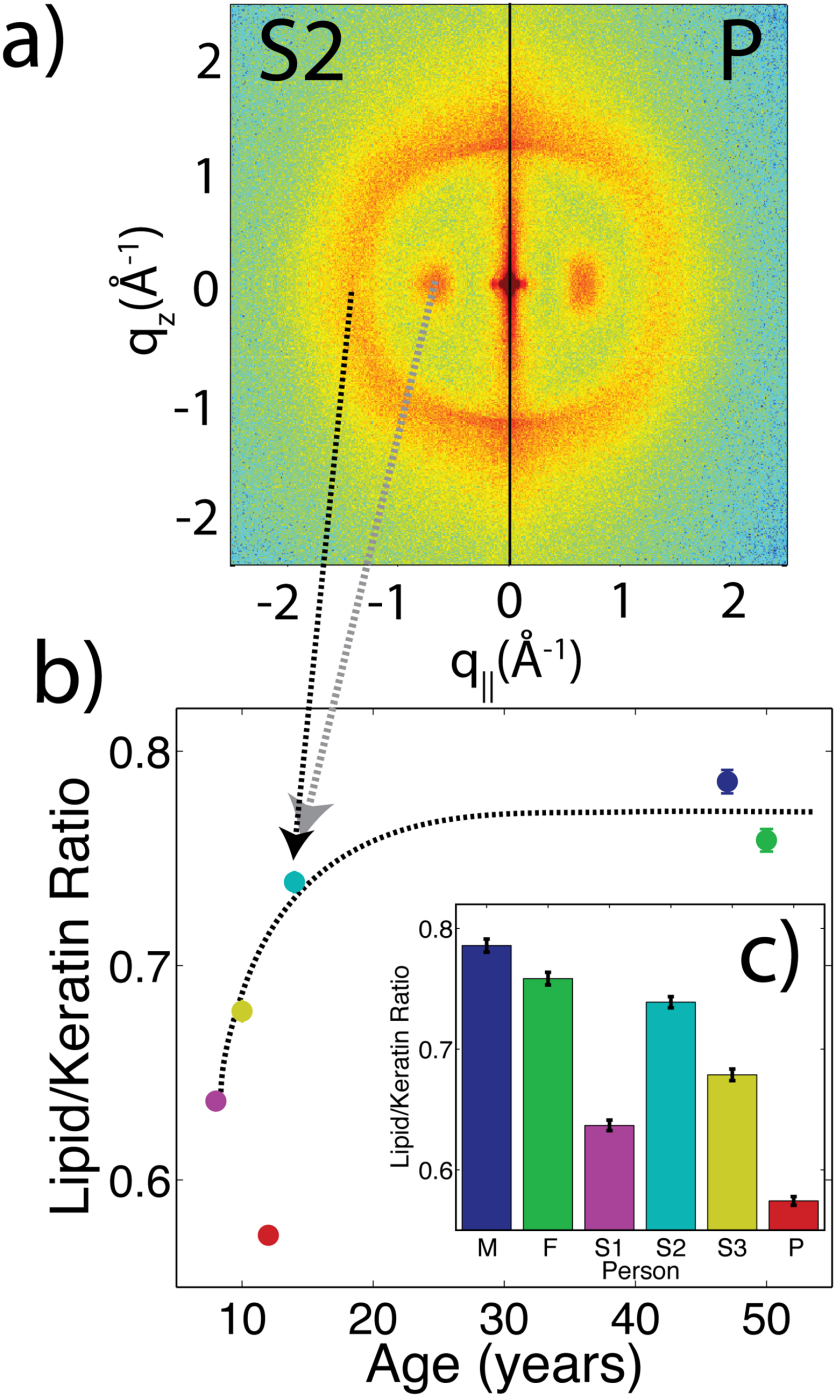
Analysis of diffraction images. a) A split-image, with the right showing the 2-dimensional pattern from P and the left showing the pattern from S2. Qualitative differences in the relative intensity of the keratin peak at 9.8 Å are visible. The keratin and lipid peaks were integrated from 0 < *ϕ* < 45° (where *ϕ* is an angle defined from the *q*_||_ axis), and from 0.55 Å^−1^ < *Q* < 0.8 Å^−1^ or 1.0 Å^−1^ < *Q* < 2.5 Å^−1^, respectively. b) The lipid/keratin ratio of intensities is shown as a function of age and c) identified by each person. The dashed-black line in b) is not a fit, but instead a guide derived from past reports on changes in lipid content in hair [14, 32, 33].

However, differences were observed in the relative intensities of the lipid and keratin peaks. Fig 7a) shows a split comparison between S2 and P. The 2-dimensional intensity maps of each sample were cut in half and combined such that the right half shows scattering from P and the left half scattering from S2.

In the 2-dimensional scan of P, the keratin peak at 9.8 Å appears more intense relative to the lipid scattering. To quantitatively test this observation, regions of the 2-dimensional plots were integrated to capture scattering from the keratin peak as well as the lipid peak. We note that the lipid and amorphous contributions could be well separated to exclude a contribution of the amorphous halo. The ratio of these two integrated intensities is proportional to the volumetric ratio of lipid and keratin in the hair fibre. The results for all family members are presented in Fig 7b). The patient has the lowest ratio of lipid/keratin. However, it is known that the lipid/keratin ratio changes also a function of age [14, 32, 33]. Fig 7c) plots lipid/keratin ratios for all family members sorted by age together with a general guide derived from past reports [14, 33]. While all family members follow the predicted trend, the patients hair has a significantly lower lipid/keratin ratio for his age.

## Discussion

Hair of a male patient, aged 12 years, with a novel ribosomopathy was tested by various biophysical techniques. One of the symptoms of the patient’s novel disorder is thin, brittle hair. Optical microscopy, tensile tests, and X-ray diffraction were combined to examine the structure and properties of the hair fibre on macroscopic and microscopic length scales. In comparing the results from his fibres to his family’s, the goal was to identify and characterize a clinical phenotype in hair for this ribosomopathy.

In general, measurements of all the hair yielded results which were within normal limits, indicating all hair samples were intact. The diameter, Young’s modulus, and molecular structure of the hair samples all agreed with previous reports [4]. However, there were subtle variations observed between the patient and his family. These differences together suggest a change to the composition of the hair, perhaps caused by the *de novo* disease.

### Differences Observed in the Patient

First of all, the hair fibre of the patient is measurably thinner. His hair is an average of 60 *μ*m in diameter, while the diameter of caucasian hair (for a teenaged male) is 70-80 *μ*m [13, 15]. Indeed, this is the diameter observed for the patient’s older brother (S2) who is also a teenaged male. His father’s hair is also closer to 55 *μ*m in diameter, however, this is expected for an older caucasian male as the hair diameter is known to peak at 70-80 *μ*m, at an age of 20-25 years for men, then decline monotonically with age [13, 15]. The average diameter of his hair was measured to be 55 *μ*m, which is in agreement with previous reports for men his age.

Secondly, X-ray diffraction measurements suggest the patient has a lowered molecular ratio of lipids relative to keratin in his hair structure. The X-ray diffraction measurements are sensitive to the molecular packing of the lipid and keratin molecules, represented by in-plane peaks at 4.5 Å and 9.8 Å. The integrated area of these two peaks is proportional to the relative content of lipid and keratin molecules, respectively. The ratio of these areas is lowest in the patient. However, the position and width of these peaks agree, suggesting that while molecular fraction of these components has changed, their structure remains the same. This result can be explained by either an increase in the amount of keratin, or decrease in the amount of lipid.

Note that like hair fibre width, the volume of lipids within the fibre is not constant with age. The volume of lipids increases rapidly at ages 8-10, then decreases again at age ∼50 [14]. The members without the ribosomopathy follow a trend observed previously (as sketched by the black line in Fig 7c)) [14, 32, 33], while the patient does not.

Lastly, the Young’s modulus of the hair is not significantly different compared to his family. In past experiments, researchers have, in general, explained the linear stretching of the hair to elastic deformations of the keratin coiled-coils within the fibre, where the keratin proteins are modelled as Hookean springs [17]. Recently, however, Chou *et al.* related the linear regime to stretching of the protein matrix in contact with the keratin macrofilaments [16]. Since keratin is responsible for the restoring force (where the strength of the force is given by the Young’s modulus), increased keratin by volume should increase the restoring force and Young’s modulus. However, as no significant increase in stiffness is observed, we conclude that the concentration of keratin remains roughly the same. We can therefore report two main findings: the patient’s hair is (1) thinner and (2) contains less lipid (and not more keratin) when compared to his family.

### Possible Relevance to Disease

The patient studied here presents with a de novo ribosomopathy, caused by a mutation on gene RPS23. The patient’s symptoms are analogous to other ribosomopathies, such as diamond-blackfan anemia (although he does not have anemia). The symptoms can be broadly classified as developmental issues, and include hypotonia, elastic skin, and learning disabilities.

While the biophysical experiments here cannot conclusively connect the properties of his hair fibre to his disease, the results of this study suggest that the development of his hair is different from his families. There is evidence his hair fibre diameter and lipid content are not at normal levels. Ribosomopathies have, in the past, presented with endocrine deficiencies [34–36]. The endocrine system is involved in hair development [37], and in particular plays a role in hair diameter and lipid content [14]. It can therefore be speculated that an altered endocrine system is responsible for the change in biophysical properties of the hair fibre.

Many diseases result in observable changes to the hair fibre, making the hair fibre useful as a diagnostic tool. The biophysical characterization of hair has already been incorporated as a diagnostic tool for breast cancer [12]. In particular, the detailed study of hair can be used to characterize clinical pathologies and inform exome sequence analysis for the diagnosis of *de novo* genetic diseases. Changes in the properties of hair in this study are likely directly related to the presence of a *de novo* mutation in the patient. While the mutation in the subject studied here is already known, in the future, these results can be used to inform diagnosis of similar diseases.

## Conclusion

We report the detailed hair pathology of a patient with a mutation to the ribosome gene RPS23. The hair structure of a patient with a *de novo* ribosomopathy was analyzed from macroscopic to molecular length scales by optical microscopy, tensile tests, and X-ray diffraction. Results from the patient were compared to his family and to literature values, in order to characterize a phenotype for this ribosomopathy.

X-ray diffraction did not reveal a change in the molecular structure of the hair, *i.e.*, the organization of keratin into coiled-coils and the structure of intermediate filaments. Visual inspection did not reveal any deformities in the hair fibre. However, diffraction results suggest the patient’s hair has 20% less lipid content. In addition, we observe the diameter of his hair is reduced by 22%. Tensile tests were performed on the hair and revealed the Young’s modulus of the fibre is roughly unchanged in the patient. The pathologies together suggest hair from the patient is subject to an altered developmental cycle caused by his ribosomopathy

The diagnosis of diseases caused by *de novo* mutations can present clinical challenges. The detailed characterization of pathologies from these diseases can inform diagnosis. A detailed pathology can be used to reduce leads in exome sequencing to find *de novo* mutations and identify similar ribosomopathies in the future.

## Materials and Methods

### Obtaining Hair Samples

This research was approved by the Hamilton Integrated Research Ethics Board (HIREB) under approval number 14-474-T. Written consent was obtained from all participating adults, and informed consent was also obtained, in writing, from the next of kin on behalf of the children enrolled in the study.

The samples size consisted of six people: the male subject or patient (aged 12 years, denoted P), his mother (47 years, denoted M); his father (50 years, denoted F); a sister, herein known as sibling 1 (8 years, S1); a brother, herein known as S2 (14 years, S2); and a second sister, herein known as S3 (10 years, S3).

At the beginning of the study, around 200 hair strands were obtained from the subject’s frontal scalp region, less than 10 cm from the roots, and mailed to Hamilton, Canada, from the Netherlands.

### Optical Microscopy of Hair Samples

High-resolution surface images of the hair samples were obtained on an Axio Observer D1 Inverted Microscope. Hairs were mounted, untreated, on to the image stage and illuminated from below. Images in Fig 1d) were obtained with 64x magnification. Images were acquired using Zeiss Zen Pro software.

**Fig 8:**
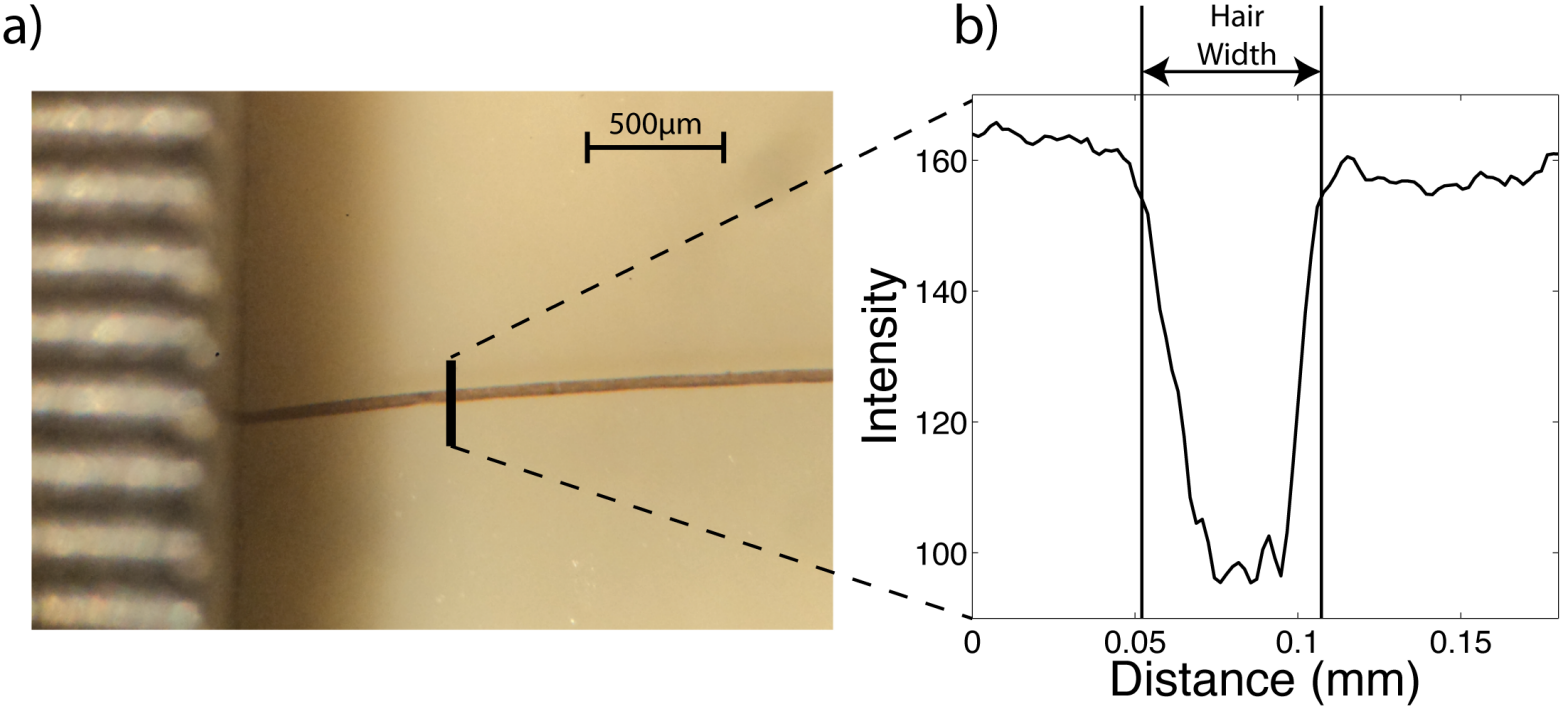
A diagram illustrating how the cross-sectional diameter of the hair is obtained. Hair samples are imaged on the Celestron 10X microscope. Using ImageJ, the intensity profile tangential to the hair axis is obtained. The width of the hair is determined from the width of the intensity profile across the hair.

Optical images of hair were also obtained on a Celestron (Model 44202) microscope with 10× widefield lens. Image were recorded using a Sony DSCW310 Cybershot digital camera. A section of hair about 6 mm long was recorded, as depicted in Fig 8a). For each image, as depicted in Fig 8b), cross sectional intensity profiles were measured tangential to the hair fibre at five points along the hair, and the width of the hair extracted using this intensity profile. The width was averaged for each image from all of the cross-sections taken. Ten hairs were averaged for each person.

### Tensile Experiments of Hair Samples

Tensile experiments were perfomed using an Instron 5943 single column testing instrument, operated by the Biointerfaces Institute at McMaster University. For performing tensile tests, the ends of the hair samples were mounted onto 1 cm × 1 cm silicon wafers. First, the hair was tied into a tight knots at either end of the hair, within 5 mm of the end of the fibre. The knotted section of the hair was placed on a silicon wafer and then epoxy was applied. A second wafer was applied to the top of the epoxy, sandwiching the hair between the wafers and fixing the knot within the epoxy. This procedure was repeated for the opposite end of the hair. The samples were allowed to dry overnight before testing. This preparation procedure was designed to prevent slipping of the hair from the mount when it is pulled. Before testing, the effective length of the hair (the length of the hair visibly exposed between the two wafer sandwiches) was recorded. Also, microscope images were taken to determine the cross sectional area of the hair.

The wafer mounts were then sandwiched between the opposing jaws of the Instron tensile tester. The force required to extend the hair a unit length (force *vs.* extension) was collected. The curves were recorded at a rate of 7% extension per minute. Measurements were taken to the point of mechanical failure, characterized by breaking of the hair. If a hair sample slipped out the epoxy at high stress, rather than fracturing, or fractured at a location within the epoxy, the measurement was not used for analysis. Experiments were performed at ambient conditions of 25°C and 50% relative humidity.

**Fig 9:**
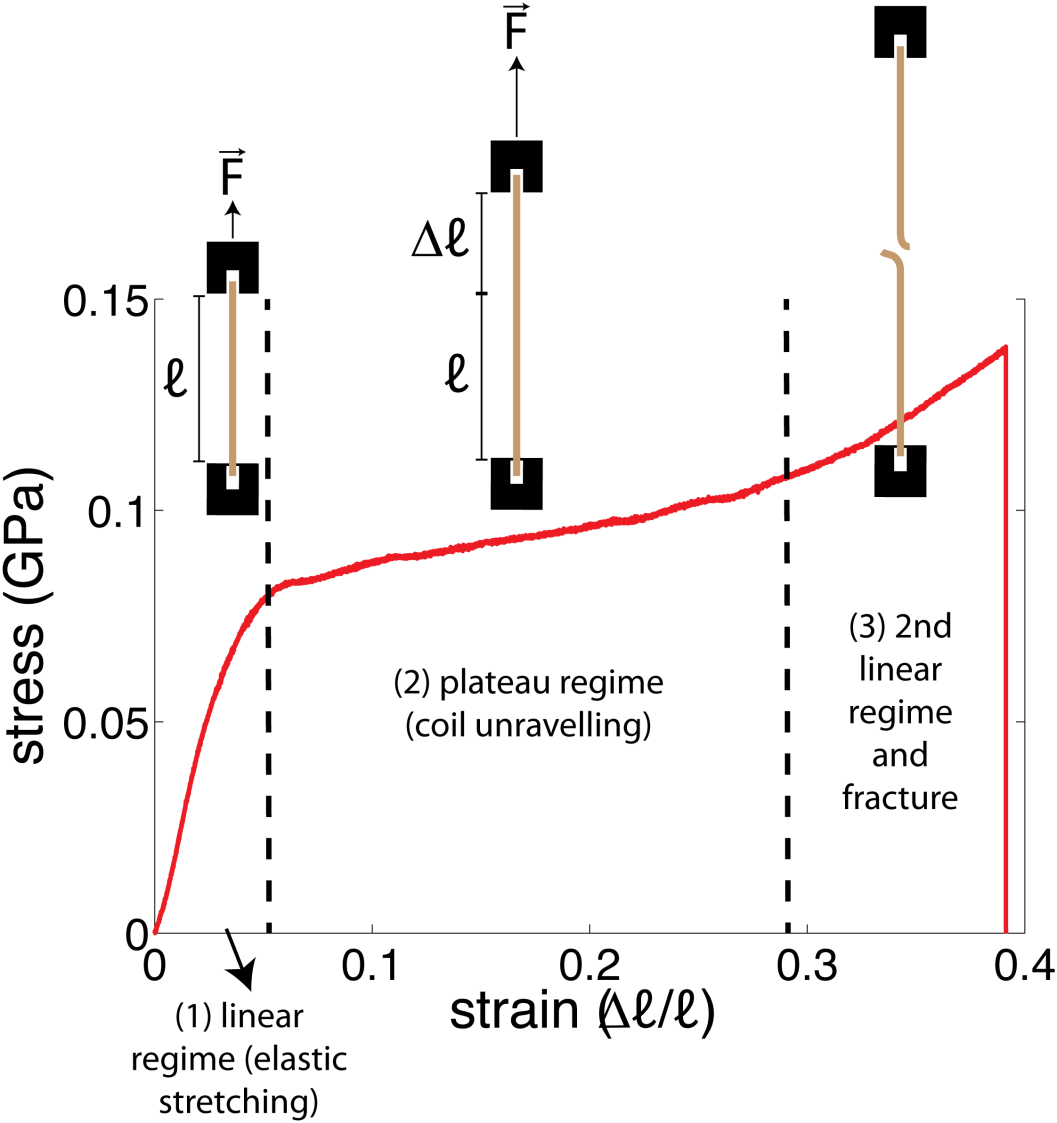
A description of the stress-strain curves obtained from tensile tests of the hair samples. 1) An initial linear regime is observed, characterized by elastic stretching of the keratin proteins in the hair; ii) A plateau region is observed, when the coiled-coil *α*-keratin is pulled into a *β*-sheet configuration; and 3) A second linear regime is observed up to the point of hair fracture [16–18, 38, 39].

Stress-strain curves were obtained by normalizing the *y*-axis of the measured force *vs.* extension curves by cross sectional area of the hair sample (obtained from microscope images) and by normalizing the *x*-axis by the length of the hair. Three distinct regions are observed in stress-strain curves of hair, as depicted in Fig 9: (1) a linear regime at <10% extension, followed by (2) a plateau at ∼10% up to ∼ 30% extension; and (3) above 30%, extension another linear regime is observed up to the point of failure [38]. The Young’s Modulus is obtained by obtaining the slope of the linear regime at low extension by a linear fit.

### Diffraction Experiments

**Fig 10:**
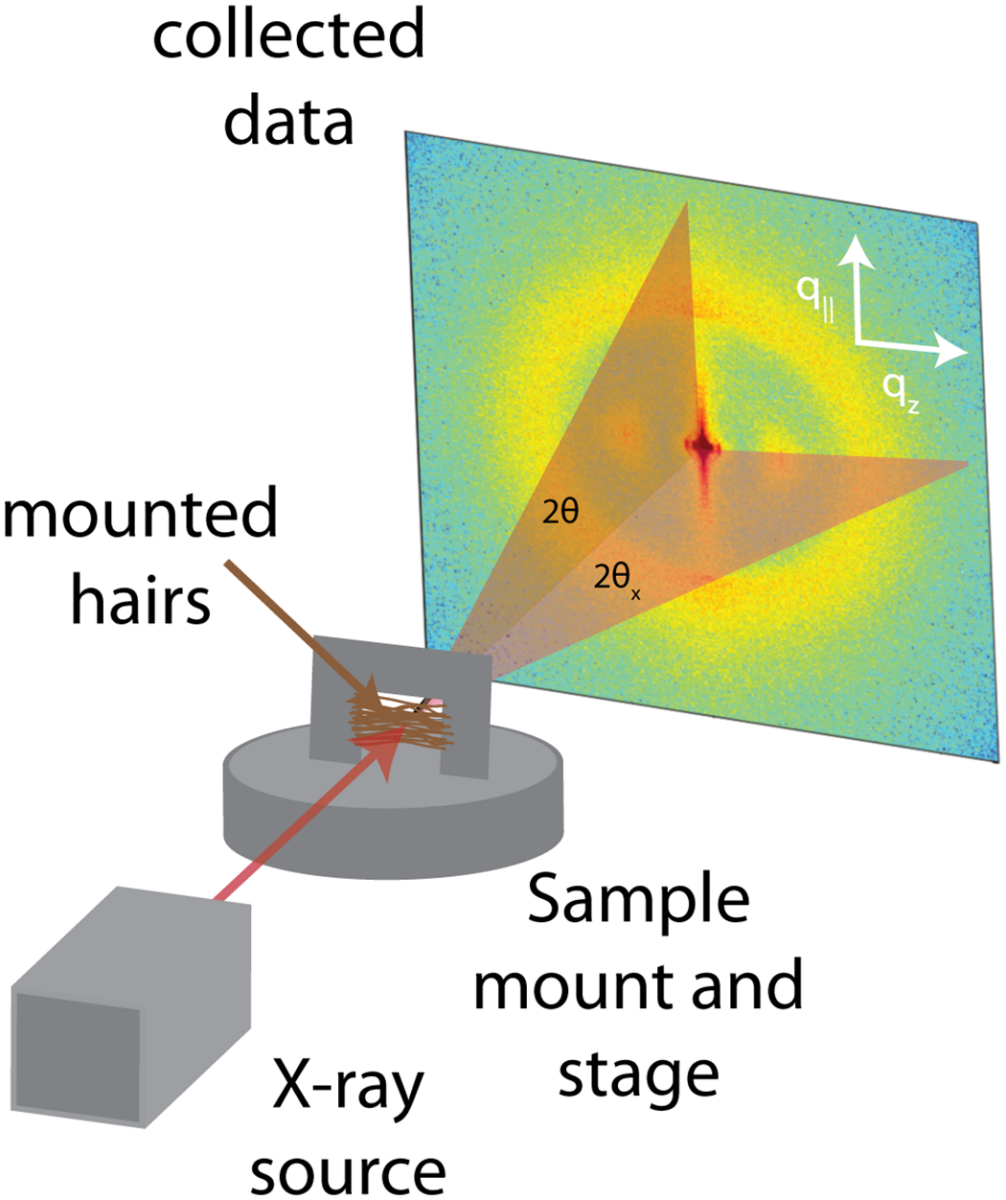
A diagram illustrating the X-ray diffraction setup for measuring the hair fibres. The hairs were cut to a length of 3 cm, and ∼50 such hairs were placed on an aluminum mount with a cut-out. The mount was fixed to the X-ray stage with sticky tack.

To prepare hair for diffraction analysis, hair samples were cut into strands ∼3 cm long. Care was taken to prevent stretching or deforming the hair strands during this process. For each subject, about 50 strands were mounted onto an aluminum apparatus, as depicted in Fig 10. The cut-out at the middle of the apparatus allows for the scattering of X-ray signals on the hair sample. The apparatus is then mounted vertically onto the loading plate of the Biological Large Angle Diffraction Experiment (BLADE) using sticky putty. All hair samples were scanned at ambient conditions, *i.e.*, a room temperature and humidity of 28°C and 50% RH.

X-ray diffraction data was obtained using BLADE in the Laboratory for Membrane and Protein Dynamics at McMaster University. BLADE uses a 9 kW (45 kV, 200 mA) CuK*α* Rigaku Smartlab rotating anode at a wavelength of 1.5418 Å. Focusing multi-layer optics provides a high intensity parallel beam with monochromatic X-ray intensities up to 10^10^ counts/(s×mm^2^). By aligning the hair strands in the X-ray diffractometer, the molecular structure along the fibre direction and perpendicular to the fibres could be determined. We refer to these components of the total scattering vector, $\vec{Q}$, as *q*_*z*_ and *q*_||_, respectively.

An illustration of *q*_*z*_ and *q*_||_ orientations is shown in Fig 10. The result of such an X-ray experiment is a 2-dimensional intensity map of a large area of the reciprocal space of -2.5 Å^−1^ < *q*_*z*_ < 2.5 Å^−1^ and -2.5 Å^−1^ < *q*_||_ < 2.5 Å^−1^. Images were taken for all hair samples in the study. The corresponding real-space length scales are determined by *d* = 2*π*/|*Q*| and cover length scales from about 2.5 to 250 Å, incorporating typical molecular dimensions and distances for primary and secondary protein structures and lipid structures. To determine keratin/lipid ratios, the intensity of the keratin peak was integrated and compared to the integrated intensity from the lipid peak. For the keratin peak, a region between 0.55 Å^−1^ < *Q* < 0.8 Å^−1^ and 0 < *ϕ* < 45° was integrated, where *ϕ* defines an angle relative to the *q*_||_ axis. For the lipid peak, the integrated area was defined by 1.0 Å^−1^ < *Q* < 2.5 Å^−1^ and 0 < *ϕ* < 45°. Note that the integration was performed only up to *ϕ* = 45° to avoid including intensity from the peak assigned to keratin along *q*_*z*_ at 5.1 Å.

## Supporting Information

S1 File Electronic Supplementary Material to: Structural Abnormalities in the Hair of a Patient with a Novel Ribosomopathy. (PDF)
