# Supplementary material for: Structural Abnormalities in the Hair of a Patient with a Novel Ribosomopathy
Source: PLoS One. 2016 Mar 16;11(3):e0149619. doi: 10.1371/journal.pone.0149619 (PMC4794122; doi:10.1371/journal.pone.0149619)
Supplement: S1 File — (PDF) [file pone.0149619.s001.pdf]

Supporting Information:
Structural Abnormalities in the Hair of a Patient with a Novel Ribosomopathy

Richard J. Alsop,[1] Asfia Soomro,[1] Yuchen Zhang,[1] Marc Pieterse,[1]
Ayodele Fatona,[2] Kimberly Dej,[3] and Maikel C. Rheinstädter*[4]

[1]*Department of Physics and Astronomy,*
McMaster University, Hamilton, Ontario, Canada

[2]*Department of Chemistry and Chemical Biology,*
McMaster University, Hamilton, Ontario, Canada

[3]*Department of Biology, McMaster University, Hamilton, Ontario, Canada*

[4]*Department of Physics and Astronomy,*
McMaster University, Hamilton, Ontario, Canada

**rheinstadter@mcmaster.ca*

Feature	M	F	S1	S2	S3	P
Keratin peak centre (Å^{-1})	0.662±0.003	0.669±0.004	0.665±0.003	0.662±0.003	0.661±0.003	0.666±0.003
Keratin peak width (Å^{-1})	0.150±0.006	0.151±0.006	0.146±0.006	0.150±0.005	0.156±0.006	0.154±0.006
1st Lipid peak centre (Å^{-1})	1.42±0.02	1.42±0.02	1.43±0.02	1.45±0.01	1.43±0.02	1.41±0.02
1st Lipid peak width (Å^{-1})	0.26±0.02	0.29±0.03	0.28±0.03	0.31±0.02	0.27±0.02	0.29±0.03
Amorphous peak centre (Å^{-1})	1.80±0.04	1.83±0.05	1.82±0.04	1.89±0.03	1.81±0.03	1.81±0.04
Amorphous peak width (Å^{-1})	0.34±0.05	0.34±0.05	0.34±0.05	0.34±0.05	0.34±0.05	0.34±0.05
2nd Lipid peak centre (Å^{-1})	2.59±0.07	2.67±0.05	2.63±0.05	2.64±0.03	2.65±0.03	2.86±0.05
2nd Lipid peak width (Å^{-1})	0.51±0.18	0.50±0.15	0.40±0.15	0.40±0.15	0.40±0.15	0.61±0.20

TABLE A. **Parameters determined from the fits to wide-angle data**. No differences in position or width of the features are observed indicative that the molecular structure of coiled-coil keratin phase and structure of intermediate filaments in identical within the experimental error between family members.
